# Methods for Improving the Straightness Accuracy of Laser Fiber-Based Collimation Measurement

**DOI:** 10.3390/s26092676

**Published:** 2026-04-25

**Authors:** Ying Zhang, Peizhi Jia, Qibo Feng, Fajia Zheng, Fei Long, Chenlong Ma, Lili Yang

**Affiliations:** 1 Key Laboratory of Luminescence and Optical Information, School of Physical Science and Engineering, Beijing Jiaotong University, Beijing 100044, China; 22110515@bjtu.edu.cn (Y.Z.); 21118036@bjtu.edu.cn (F.L.); 23115198@bjtu.edu.cn (C.M.); 25110634@bjtu.edu.cn (L.Y.); 2 Beijing Aerospace Institute for Metrology and Measurement Technology, Beijing 100076, China; jiapeizh@126.com

**Keywords:** straightness measurement, quadrant detector, laser collimation, beam drift compensation, air turbulence

## Abstract

Laser fiber-based collimation straightness measurement can eliminate the intrinsic drift of the laser source while offering a simple configuration and simultaneous measurement of straightness in two orthogonal directions. As a high-precision optoelectronic sensing method, it has been widely used for the measurement of straightness, parallelism, perpendicularity, and multi-degree-of-freedom geometric errors. However, two common issues remain in practical applications. One is the nonlinear response of the four-quadrant detector, the core position-sensitive sensor, which is caused by detector nonuniformity and the quasi-Gaussian distribution of the spot. The other is the degradation of measurement performance by atmospheric inhomogeneity and air turbulence along the optical path, particularly in long-distance measurements. To address these issues, a two-dimensional planar calibration method is first proposed to replace conventional one-dimensional linear calibration. A polynomial surface-fitting model is introduced to correct the nonlinear response and inter-axis coupling errors of the four-quadrant photoelectric sensor. Simulation and experimental results show that the proposed method significantly reduces the standard deviation of calibration residuals and improves measurement accuracy. In addition, based on our previously developed common-path beam-drift digital compensation method, comparative experiments were carried out on double-pass common-path and single-pass optical configurations employing corner-cube retroreflectors, and theoretical simulations were performed to analyze the influence of air-turbulence disturbances on measurement stability. Both theoretical and experimental results show that the double-pass common-path configuration exhibits more pronounced temporal drift. Therefore, a real-time digital compensation method for beam drift in long-distance single-pass common-path measurements is proposed. Experimental results demonstrate that the proposed method effectively suppresses drift induced by environmental air turbulence and thereby improving the accuracy and stability of long-travel geometric-error and related straightness measurement for machine-tool linear axes.

## 1. Introduction

The laser fiber-based collimation straightness measurements can effectively eliminate the intrinsic drift of the laser source and offer advantages such as a simple system configuration and the capability to simultaneously measure straightness in two orthogonal directions. Therefore, it has been widely applied to the measurement of straightness, perpendicularity, and other forms and position errors in precision machine tool guideways, coordinate-measuring machines, and related equipment. The basic principle is to use a laser beam as a straight reference line to measure straightness errors, from which other geometric errors can be derived [1]. In practical applications, the travel ranges of the guideways for most machine tools are generally within one meter. However, for some large-scale gantry machine tools, the guideway length can be several meters, or even extend to 10 m. For such long-distance measurement requirements, laser-based collimation methods offer distinct advantages over approaches relying on physical straightness standards, as they are noncontact, exhibit high dynamic response, and are well suited for long-range measurements [2]. Owing to their fast response, high resolution, simple signal processing, and the ability to provide two-dimensional (2D) position outputs, four-quadrant detectors (QDs) have been extensively used for signal detection in laser fiber collimation systems [3]. In recent years, high-precision optoelectronic sensing and advanced optical metrology have developed rapidly, showing clear trends toward compactness, real-time operation, and high sensitivity [4,5,6], which further reflects the frontier progress in advanced optical sensing and precision measurement technologies. However, in laser fiber collimation methods for long-distance straightness measurement of machine tool linear guideways, beam drift remains a key factor restricting further improvement in measurement accuracy. It significantly degrades the accuracy of laser collimation measurements, making it difficult to satisfy the precision requirements of long-distance guideway measurement. Therefore, beam drift has become an important issue that must be addressed urgently.

One of the major factors affecting the accuracy of the laser fiber-based collimation measurements is the nonlinear error of the QD. In practical applications, the quasi-Gaussian intensity distribution of the laser spot, combined with the inherent nonuniform response of the sensor, inevitably leads to nonlinear output characteristics of the QD. This nonlinearity becomes more pronounced in long-range straightness measurements [7], significantly reducing both the measurement accuracy and effective measurement range. To address this issue, many researchers have proposed methods to mitigate the nonlinear behavior of QDs. For example, Zhang et al. [8] employed a piecewise polynomial fitting algorithm to extend the linear measurement range of a sensor and effectively reduce nonlinear errors. Other studies have introduced machine learning and neural network techniques to establish mapping models between sensor outputs and spot positions, enabling the compensation of errors that are difficult to correct using conventional algorithms and achieving higher positioning accuracy [3,9]. In addition, some studies focused on the weak signal and low signal-to-noise ratio issues of QDs in noisy environments, proposing improved signal-processing algorithms and error compensation models. Wang et al. [10] introduced gap-size correction and error compensation factors in free-space optical communication systems, significantly improving the spot position detection accuracy under weak-signal conditions; however, most of the aforementioned approaches are still based on one-dimensional (1D) calibration methods.

Beam drift is another dominant factor that degrades the accuracy of the laser fiber collimation straightness measurements, particularly in long-distance applications [11,12]. For long-range, high-precision straightness measurements, effective suppression and compensation of beam drift are essential. Beam drifts can be classified into three categories. The first is mechanical drift of the measurement structure (including the measurement head and target mirror), which manifests as a slow time-varying behavior; this type of drift can be largely eliminated by employing common-path beam drift compensation methods [13]. The second category is the inherent parallel and angular drift of the laser source itself. Both theoretical analyses and experimental studies have shown that coupling the laser to a single-mode fiber (SMF), especially when using semiconductor lasers with fiber pigtails, can significantly suppress the intrinsic parallel and angular drift [14]. The third category originates from air turbulence and inconsistencies in environmental parameters along the optical path, which leads to beam wander and bending [11,15]. Reducing the influence of this type of beam drift on laser fiber-based collimation measurement has become a key research focus in this field. Among these, the present work mainly addresses mechanically induced drift and environmentally induced beam-path deviation/beam drift, together with their influence on long-distance measurement and the corresponding compensation methods. The source-inherent pointing drift is largely suppressed by the use of single-mode fiber coupling and is therefore not the main focus of this study. Although numerous methods have been reported, this problem has not yet been fully resolved. In general, approaches for mitigating the effects of air turbulence and environmental variations on collimation measurements can be broadly classified into two categories: beam-drift suppression methods and beam-drift compensation methods [15].

Beam-drift suppression methods aim to reduce the laser beam drift at its source and along the propagation path. Environmental control and passive shielding are important approaches for mitigating beam drift. During their propagation in air, laser beams are inevitably affected by fluctuations in the refractive index and atmospheric turbulence [1]. To address this issue, researchers have designed physical shielding devices to stabilize the optical-path environment. Liu et al. [16] proposed the installation of a “turbulence shielding cover” along the optical path to attenuate the disturbance of atmospheric turbulence on the laser beam. The experimental results demonstrated that this approach can reduce the measurement noise induced by air disturbances by approximately 90%. Similarly, Li et al. [17] enclosed a laser propagation path with corrugated flexible hoses and employed an air pump to circulate and regulate the stability of the refractive index inside the tube, thereby significantly reducing beam drift. This scheme improves the positional stability of the laser spot by more than 79%, providing a low-cost solution for enhancing the measurement stability. However, in many practical scenarios, such methods are difficult to implement because the optical path cannot be fully enclosed or shielded.

Beam drift compensation methods can be broadly classified into active and passive. Active compensation methods involve real-time monitoring of beam drift and compensation through a feedback-control system that adjusts the beam position and direction. For example, Zhao et al. [18] suppressed the linear and angular drift of a laser beam by applying intensity modulation and SMF-based pre-alignment combined with fast feedback control based on the detected drift amplitude. Huang et al. [19] employed a 2D hybrid mirror angle steering mount that enables both large-angle steering for optical axis alignment and fine angular adjustment driven by piezoelectric actuators for real-time compensation of beam angular drift. In contrast, passive compensation methods compensate for the influence of beam drift on straightness or small-angle measurements through data processing based on the real-time monitoring of laser beam drift. Feng et al. [13] proposed a common-path laser beam drift digital compensation method and applied it to the four-degree-of-freedom error measurement of linear guideways, effectively reducing the influence of beam drift on the straightness, pitch, and yaw angle measurements. Chen et al. [15] also developed a heterodyne interferometric straightness measurement system with laser drift compensation to achieve sub-micrometer straightness measurement accuracy over a linear motion range of up to 4 m. In addition, some studies have numerically compensated for the optical path bending drift caused by a nonuniform air refractive index by establishing environmental models. Long et al. [20] proposed a dual-wavelength laser-based compensation method for air-turbulence-induced beam deviation in a 6-DOF simultaneous measurement system. By establishing a dual-wavelength proportional cancellation model, the influence of air turbulence on beam deviation was effectively reduced. For instance, You et al. [21] used finite element thermal field analysis to compensate for laser beam deflection induced by air refractive index gradients, and the experimental results confirmed improved straightness measurement accuracy in both the horizontal and vertical directions after compensation. Hu et al. [22] designed a direction measurement module based on a dual position-sensitive detector (PSD) to measure the directional deviation of the incident beam in real time, thereby correcting the straightness and displacement errors through a corresponding mode. However, existing beam-drift suppression and compensation methods still have certain limitations in practical applications. Environmental suppression methods usually rely on enclosed optical paths or shielding conditions, which are difficult to implement in some open or on-site measurement scenarios. Active compensation methods, by contrast, often require additional actuators and feedback control systems, resulting in increased system complexity and implementation cost. In comparison, the method proposed in this paper does not require complex active control, features a simpler system structure and lower cost, and is therefore more suitable for engineering applications in long-distance straightness measurement.

Therefore, based on a previous study [13], this paper proposes a single-pass common-path laser beam drift compensation method. Both the theoretical analysis and experimental results demonstrate that the proposed method can effectively suppress the influence of beam drift on the straightness measurement accuracy. To address the error increase in long-distance straightness measurement for precision machine-tool linear axes and long-travel linear stages, this paper further incorporates a two-dimensional calibration method for the quadrant detector, so that environment-induced drift errors as well as the detector nonlinearity and coupling errors can be compensated simultaneously, thereby improving the accuracy and stability of bidirectional straightness measurement under long-distance conditions.

## 2. Laser Fiber-Based Collimation Straightness Measurement with Common-Path Digital Beam Drift Compensation

**Figure 1:**
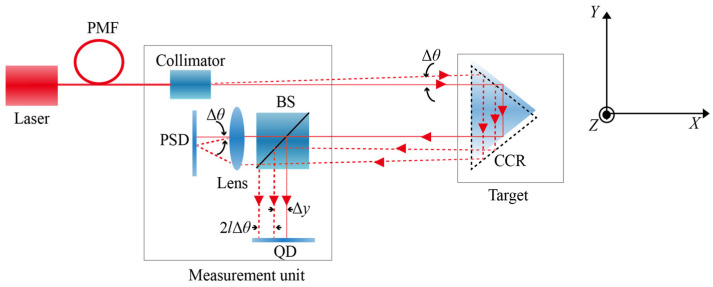
Schematic of the double-pass optical path with drift compensation.

The laser fiber-based collimation straightness measurement method is illustrated in Figure 1 [13]. The laser beam is reflected by a corner cube retroreflector (CCR) and returned to the measurement head, where it is detected by a QD. A direct correspondence exists between the motion of the sensor and target mirror with respect to straightness. Within a relatively small measurement range, this relationship can be expressed by the position of the laser spot on the QD as follows:

(1) $$\begin{array}{l} \Delta y=\frac{\Delta Y_{\mathrm{QD}}}{2}\\ \Delta z=\frac{\Delta Z_{\mathrm{QD}}}{2} \end{array},$$

where Δ*y* and Δ*z* denote the horizontal and vertical straightness errors, respectively; Δ*Y*_QD_ and Δ*Z*_QD_ represent the horizontal and vertical position variations in the laser spot on the QD.

As shown in Figure 1, a polarization-maintaining fiber (PMF) was employed to preserve the polarization state of the laser beam during fiber delivery, after being reflected by a CCR, the laser beam was split into two beams by a beam splitter (BS). One beam was transmitted through the BS and directed to a PSD to monitor the angular drift of the laser beam, whereas the other beam was reflected by the BS and incident on a QD for straightness error measurement. As both beams share an identical optical path after reflection by the CCR before reaching the measurement head, the common-mode drift induced by various factors can be effectively suppressed.

Based on the angular drift Δ*θ*, measured by the PSD, the additional linear displacement caused by beam angular drift can be approximated as ±2*l*Δ*θ*, where the sign depends on the actual measurement configuration and *l* is the distance between the measurement head and the target mirror. Consequently, the true straightness error after the beam drift compensation can be expressed as follows:

(2) $$\Delta y=\frac{\Delta Y_{\mathrm{QD}}}{2}\pm l\Delta \theta ,$$

Similarly, the vertical straightness error after compensation for the angular drift Δ*β* can be expressed as

(3) $$\Delta z=\frac{\Delta Z_{\mathrm{QD}}}{2}\pm l\Delta \beta ,$$

By applying digital compensation, this method can effectively suppress drift errors induced by laser source drift, mechanical drift, and air turbulence, thereby enabling high-precision straightness measurements. This approach is referred to as the common-path digital compensation method for laser beam drift.

## 3. Two-Dimensional Planar Calibration and Nonlinear Error Correction of the Quadrant Detector

**Figure 2:**
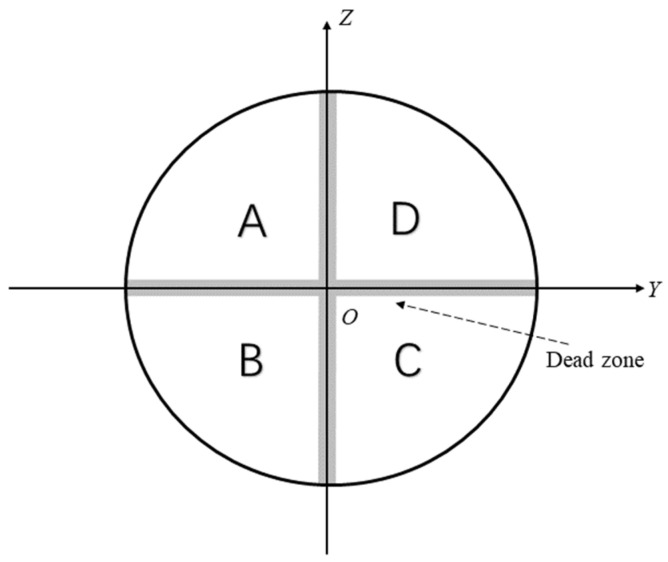
Schematic illustration of the four-quadrant photodiode and the arrangement of quadrants A, B, C, and D. Point *O* denotes the origin, and the central gap indicates the dead zone between adjacent quadrants.

A QD detects the intensity distribution of a laser spot using four quadrant photodiodes and determines the position of the spot center based on the proportional relationships among the output photocurrents of the quadrants [23], the measurement principle of which relies on the energy distribution of the laser spot over the quadrants. The spot center position is obtained by calculating the differences between the photocurrents of the quadrants. The photocurrents generated in the quadrants are denoted as *I*_A_, *I*_B_, *I*_C_, and *I*_D_, as illustrated in Figure 2. Accordingly, the position of the laser spot center can then be calculated using Equation (4) [24,25]:

(4) $$\begin{array}{l} \Delta Y_{\mathrm{QD}}=k\frac{\left(I_{A}+I_{B}\right)-\left(I_{C}+I_{D}\right)}{I_{A}+I_{B}+I_{C}+I_{D}}\\ \Delta Z_{\mathrm{QD}}=k\frac{\left(I_{A}+I_{D}\right)-\left(I_{B}+I_{C}\right)}{I_{A}+I_{B}+I_{C}+I_{D}} \end{array},$$

where *k* is a proportionality coefficient. Ideally, the calculated coordinates obtained from the QD output should exhibit a linear relationship with the actual spot position, and the *Y*- and *Z*-directions should be mutually independent. However, in practice, significant nonlinear distortion exists between the sensor output signals and the true spot position owing to factors such as nonuniform gain among the quadrant elements, coupling effects arising from the quasi-Gaussian intensity distribution of the laser beam, and the presence of dead zones between adjacent quadrants [4]. Conventional 1D calibration methods typically move the laser spot separately along the horizontal or vertical directions and fit the relationship between the detector output and spot position through line scanning [7]. Such 1D calibration approaches suffer from inherent nonlinear errors and inter-axis coupling errors caused by sensor installation misalignments [26]. To overcome these limitations, a 2D planar calibration method was proposed, in which the QD was calibrated using grid-based sampling over the entire detection plane. A third-order 2D polynomial model was employed to perform planar calibration of the QD, thereby correcting the coupling between the horizontal and vertical directions.

**Figure 3:**
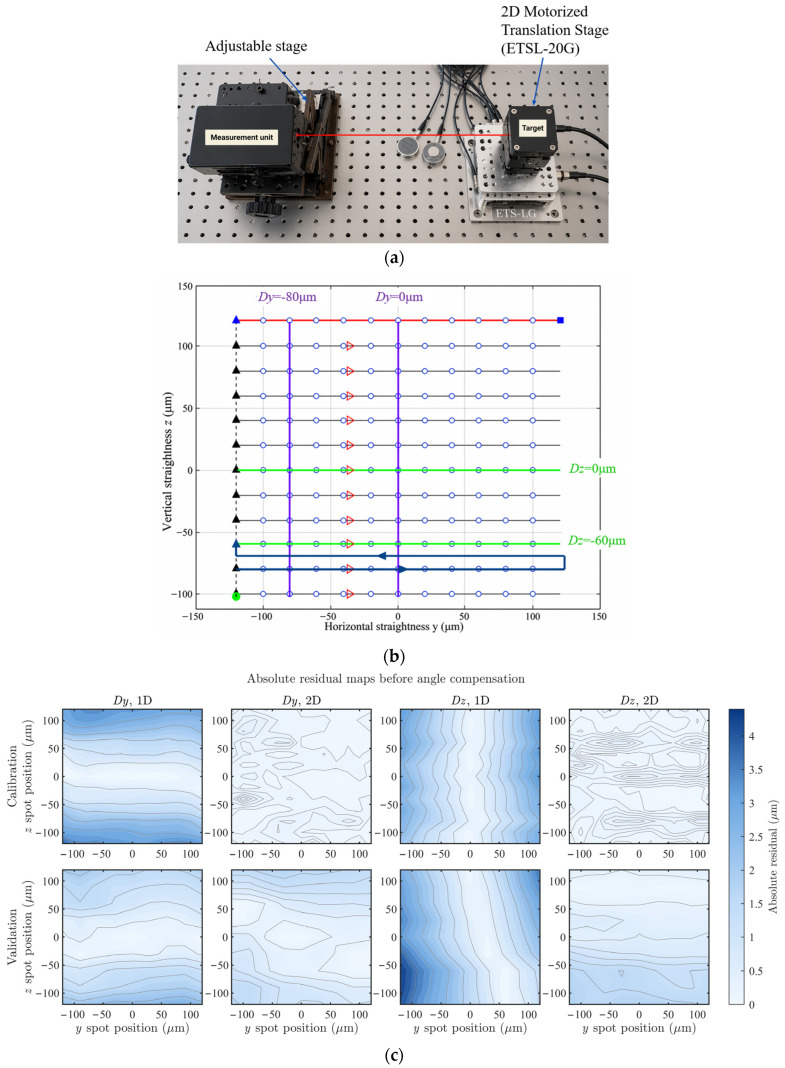
The proposed 2D calibration method: (**a**) Photograph of the 2D calibration experimental setup. (**b**) Trajectory diagram of the 2D calibration scanning, where the colored lines indicate the representative scanning paths selected for subsequent comparison, including *D*y = −80 μm, *Dy* = 0 μm, *D*z= 0 μm, and *D*z = −60 μm. (**c**) Absolute residual maps before angle compensation for the 1D and 2D calibration models in the *Dy* and *Dz* directions, for both calibration and validation datasets.

An experimental photograph and the 2D calibration scanning procedure of the QD-based sensing system are shown in Figure 3a,b, respectively. To obtain highly accurate and well-controlled reference displacements, an ETSL-20G motorized translation stage (Sanying MotionControl Instruments Ltd., Tianjin, China) was employed as the calibration standard. The stage provides a resolution of 0.1 µm, with bidirectional repeatability of ±0.2 µm along both the *Y* and *Z* axes. A grid-based scanning was performed within a range of ±120 µm, and datasets with different step sizes were used separately for model fitting and validation. The resulting 2D calibration outcomes are presented in Figure 3c, which illustrates the absolute residual maps of the horizontal *Dy* and vertical *Dz* straightness for both the calibration and validation datasets of the sensor. The horizontal and vertical axes denote the reference straightness displacements in the *Y*- and *Z*-directions, respectively, and the color scale represents the absolute residual magnitude. The upper row shows the calibration results, whereas the lower row shows the validation results.

**Figure 4:**
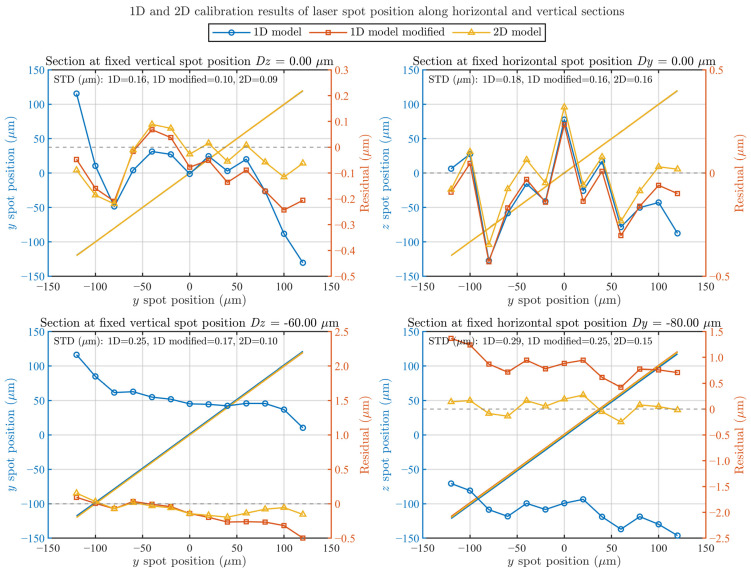
Comparison of calibration results and residuals for selected horizontal and vertical laser spot position sections. The grey dashed line indicates the zero-residual reference.

To comparatively analyze the performance of 1D linear calibration and 2D planar calibration, representative lines *Dz* = 0, *Dz* = −60 μm, *Dy* = 0, and *Dy* = −80 μm were selected from the scanning paths, as shown in Figure 3c. The corresponding horizontal and vertical cross-sections were extracted from Figure 3c as the results of the original 1D sensor calibration, referred to as the 1D model. On this basis, the inter-axis coupling errors caused by the angular misalignment of the QD were further corrected, yielding a modified 1D calibration result, denoted as the 1D model modified. These results were then compared with those obtained using the 2D calibration method, denoted as the 2D model, as shown in Figure 3. Specifically, Figure 4 presents the straightness profiles and corresponding residuals for four representative scan lines obtained using the 1D model, the modified 1D model, and the 2D model. The fitted straightness profiles are shown as straight lines referenced to the left *y*-axis, whereas the residual errors are plotted as curves with markers referenced to the right *y*-axis. The standard deviation (STD) of each residual curve is also included for quantitative comparison.

From the combined results shown in Figure 3c and Figure 4, it can be observed that compared with the conventional 1D calibration approach, the proposed 2D calibration method for the sensor exhibits the following two significant advantages:

1. The 2D calibration exhibits smaller calibration errors and provides consistent performance over the entire straightness measurement plane. As can be calculated from Figure 4, the standard deviations obtained using 1D calibration along the *Y*- and *Z*-directions are 2.10 and 1.89 µm, respectively, whereas those achieved by the 2D calibration are significantly reduced to 0.16 and 0.16 µm. In the corresponding measurement experiments, the standard deviations decrease from 1.37 µm and 2.12 µm for the 1D calibration to 0.90 µm and 0.69 µm, respectively. For the *Dy* calibration, the maximum residual of the 1D method is approximately 3.99 µm, whereas it reduced to 0.57 µm for the 2D method. Similarly, for the *D*z calibration, the maximum residual decreases from approximately 3.42 µm with 1D calibration to 0.37 µm with 2D calibration. These results demonstrate that the 2D calibration significantly improved the nonlinear characteristics of the sensor response, thereby substantially reducing the residual error level. As can also be seen from the residual maps in Figure 3c, the large-scale spatial gradients observed in the 1D calibration results are effectively suppressed after 2D calibration, leading to a more uniform residual distribution over the calibrated plane.
2. The calibration model involved in 2D calibration incorporates polynomial coupling terms, which can automatically eliminate inter-axis coupling errors caused by the angular misalignment of the sensor installation. The intuitive reason is that, when the sensor coordinate frame undergoes a small angular rotation with respect to the ideal reference frame, an ideal displacement along *Y*- will introduce a *Z*-related crosstalk component in the measured signal (and vice versa). The cross terms in the 2D model (e.g., (*xy*) and linear/quadratic terms involving the opposite-axis variable) are effectively equivalent to modeling and fitting the channel mixing in the sensor outputs induced by such a coordinate rotation, thereby enabling compensation to be achieved during calibration. In the proposed 2D calibration method, the reconstructed coordinates are obtained from *Y* = *f*_Y_(Δ*Y*_QD_, Δ*Z*_QD_), *Z* = *f_Z_*(Δ*Y*_QD_, Δ*Z*_QD_),where *g_Y_*(Δ*Y*_QD_) and *g_Z_*(Δ*Z*_QD_) are one-dimensional fitted functions, and *f_Y_*(·) and *f_Z_*(·) are third-order bivariate polynomial functions. The mixed terms in the latter explicitly account for the coupling between the two channels. In contrast, 1D calibration typically establishes independent single-axis mappings for the sensor, which are insufficient to represent inter-channel mixing; consequently, an additional axis-angle estimation followed by coordinate-rotation compensation is required to compensate for the inter-axis coupling error.

As shown in Figure 3c, the regression model constructed from the 2D calibration data indicated that the angular error in sensor installation was approximately 1.75°. Before angular correction, the standard deviations of the 1D calibration are 2.10 µm and 1.89 µm in *Y*- and *Z*-directions, respectively. After angular correction, these values are reduced to 0.21 µm and 0.77 µm. By comparison, the 2D model can inherently eliminate inter-axis coupling without any explicit angular compensation, achieving a calibration performance comparable to or even better than that of the angle-corrected 1D calibration. Specifically, the standard deviations of the 2D calibration in both the *Y*- and *Z*-directions were as low as 0.16 µm.

In summary, the proposed 2D calibration method not only significantly reduces the fitting residuals, but also automatically compensates for sensor installation angular errors while maintaining uniform calibration accuracy over the entire 2D measurement range. Consequently, it is more suitable for laser straightness measurement systems requiring high stability and precision.

## 4. Analysis of the Influence of Atmospheric Turbulence on Laser Fiber-Based Collimation Measurements in Single-Pass and Double-Pass Optical Paths

**Figure 5:**
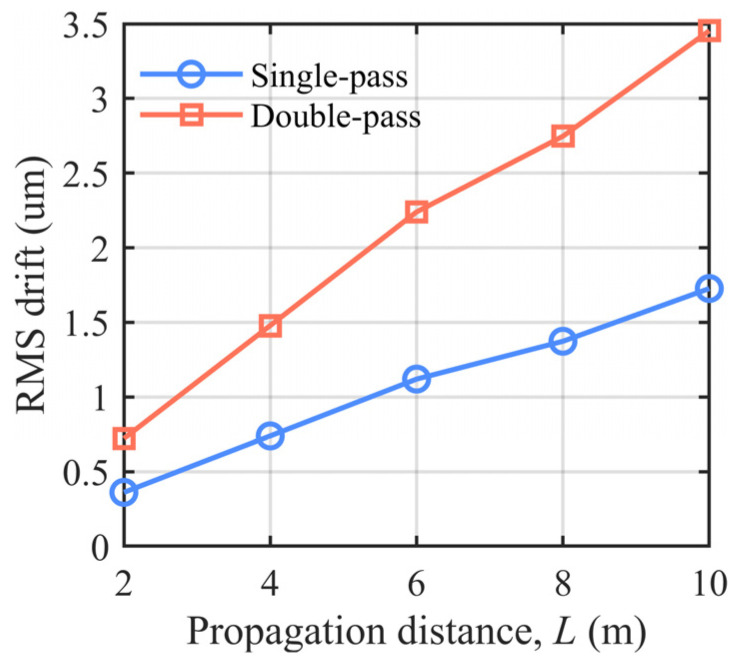
Calculated drift results for single-pass and double-pass optical paths.

When a laser beam propagates through the atmosphere, variations in the temperature, humidity, and pressure induce small fluctuations in the air refractive index, resulting in random beam deflection and jitter, commonly referred to as atmospheric turbulence. For short-distance measurements, this effect is relatively small and can be neglected [16]. However, for long-distance measurements, turbulence-induced beam wandering is a dominant factor limiting the accuracy of laser fiber-based collimation measurements [12]. To characterize the statistical properties of the air refractive index fluctuations during propagation, a non-Kolmogorov three-dimensional turbulence power spectrum was introduced [27]:

(5) $$\Phi_{n}\left(\alpha ,\kappa \right)=A\left(\alpha \right){\tilde{C}}_{n}^{2}\left(\alpha ,z\right)\kappa^{-\alpha },$$

where ${\tilde{C}}_{n}^{2}(\alpha ,z)$ denotes the non-Kolmogorov refractive-index structure constant along the laser propagation path, whereas z and *α* represent the spectral parameters associated with the power-law exponent. *A*(*α*) is a generalized amplitude function that ensures consistency between the structure function and the power spectrum. The phase screens were generated using the FFT-based spectral inversion method [27]

(6) $$\phi \left(x,y\right)=\sum_{\kappa_{x}}\sum_{\kappa_{y}}h\left(\kappa_{x},\kappa_{y}\right)\sqrt{F_{\phi }\left(\kappa_{x},\kappa_{y}\right)}\exp\left[j\left(\kappa_{x}x+\kappa_{y}y\right)\right]{\Delta \kappa }_{x}{\Delta \kappa }_{y},$$

where $h\left(\kappa_{x},\kappa_{y}\right)$ denotes a complex Gaussian random variable with zero mean and $F_{\phi }\left(\kappa_{x},\kappa_{y}\right)=0.023C_{n}^{2}{\left(\kappa_{x}^{2}+\kappa_{y}^{2}\right)}^{-11/6}$ is defined as a power spectral density function that conforms to the Kolmogorov spectrum determined by atmospheric refractive-index fluctuations [2]. Figure 5 shows the MATLAB R2024b (MathWorks, Natick, MA, USA) simulation results obtained under a nonfrozen turbulence model, illustrating the variation in laser spot displacement with optical path length for the single- and double-pass propagation configurations, where *L* denotes the optical path length. To clearly illustrate how the drift root-mean-square (RMS) values in Figure 5 are obtained, a representative propagation distance *L* is considered. First, phase screens are generated according to the turbulence power spectrum given by Equations (5) and (6), and the beam propagation for the single-pass and double-pass configurations is simulated. Then, for each turbulence realization, the beam centroid (*x_c_*, *y_c_*) on the receiver plane is calculated from the intensity distribution *I*(*x*, *y*; *L*). Repeating this procedure *N* times at the same distance *L* yields a set of centroid drift samples ${\left\{(x_{i},y_{i})\right\}}_{i=1}^{N}$. The drift RMS at this distance is finally evaluated by the second-order statistics in Equation (4), using $x_{i}=x_{i}-\bar{x};\Delta y_{i}=y_{i}-\bar{y}$. By varying L and repeating the above steps, the “RMS drift versus propagation distance” curves shown in Figure 5 are obtained. It should be noted that the more pronounced drift observed in the dual-path common-path configuration is mainly attributed to the increased effective propagation distance rather than to additional motion of the corner-cube retroreflector (CCR). In the present experiments, the CCR remained stationary during the measurement process and therefore did not introduce extra mechanically induced drift. Although previous studies have shown that a CCR can modify the polarization state of the reflected beam, the straightness measurement in this work is based on the detected spot energy distribution and centroid displacement, without intentional polarization-selective detection [28]. Therefore, the polarization effect of the CCR is not considered the dominant source of the observed long-term drift. Instead, the dual-path configuration effectively doubles the air-perturbed propagation path, making the beam more susceptible to turbulence-induced beam wander, refractive-index fluctuations, and local airflow disturbances. This interpretation is consistent with classical beam-wander theory, which shows that the variance of beam-centroid displacement increases with propagation distance in a turbulent medium [29]. The results indicate that the standard deviation of the beam drift in the single-pass optical path was consistently smaller than that in the double-pass optical path after corner-cube reflection. This demonstrates that under identical disturbance conditions, the double-pass configuration accumulates more pronounced wavefront aberrations and angular drift owing to round-trip propagation. Furthermore, as the optical path length increased, the difference in drift between the two configurations continued to increase, indicating that the double-pass structure was more sensitive to the propagation distance and exhibited a more significant amplification of drift errors.

## 5. Comparative Experimental Study on the Influence of Air Disturbances on Laser Fiber-Based Collimation Measurements

**Figure 6:**
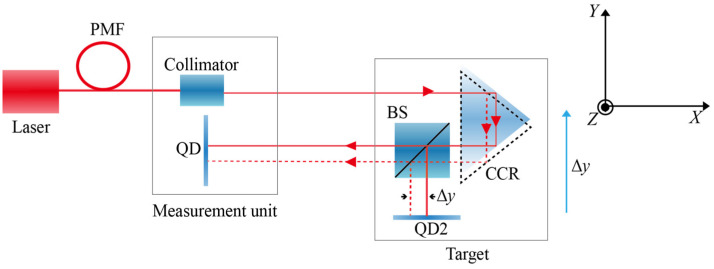
Optical configuration of the comparative experiment on the influence of air disturbances on straightness measurement.

To investigate the specific influence of atmospheric disturbances on the stability of laser straightness measurements, two optical measurement configurations were designed for comparative experiments, as shown in Figure 6. The emitted laser beam was split into two paths using a beam splitter. One path was reflected by a corner-cube retroreflector (CCR) and returned to the measurement head, where it was detected by a near-end QD, forming a double-pass optical path. The other path was directly split by BS2 and was incident on QD2, forming a single-pass optical path configuration.

**Figure 7:**
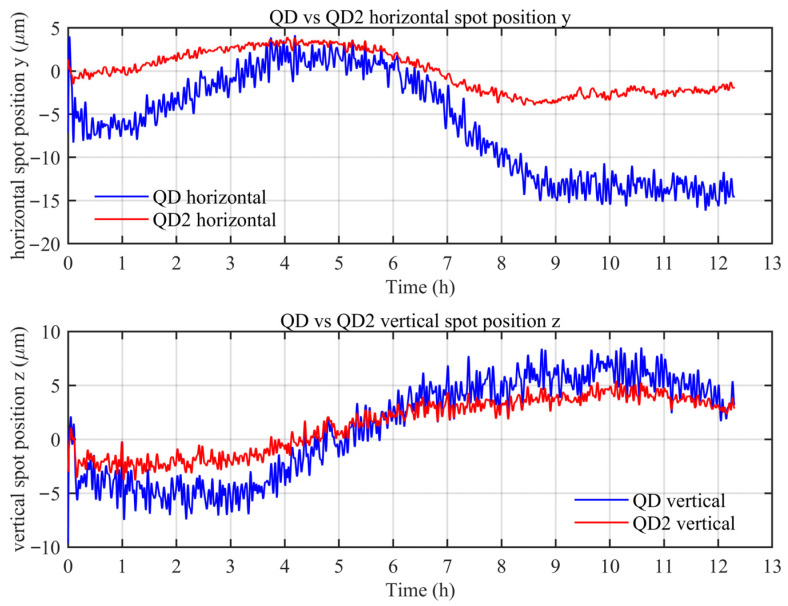
Results of the 12 h stability experiment for straightness measurement.

Based on the six-degrees-of-freedom simultaneous error measurement system developed by our research group, an experimental setup was established. The comparative experiments were conducted in an open laboratory environment on an optical platform, which does not fully reproduce the complexity of atmospheric disturbances in the actual measurement environment, but provides a controlled basis for comparing the relative susceptibility of the single-pass and double-pass configurations to air disturbances. For reproducibility, the key devices used in the experiments are specified as follows: the laser source was a Keysight 5517D laser system (Keysight Technologies, Santa Rosa, CA, USA), the QD was a First Sensor QP50-6-18U-TO8 four-quadrant detector, and the PSD was a First Sensor DL100-7-SMD position-sensitive detector (First Sensor AG, Berlin, Germany). The measurement head and target mirror were fixed to an optical platform at a separation distance of 3.1 m. A 12 h stability experiment was conducted, and the results are shown in Figure 7.

As shown in Figure 7, the data were sampled at 1 Hz. To suppress impulsive noise and high-frequency fluctuations in the measurement signals, a 9 s median filter was first applied, followed by a fourth-order Butterworth low-pass filter with a cutoff frequency of 1/180 Hz. The filtering was implemented using the MATLAB R2024b (MathWorks, Natick, MA, USA) function filtfilt to achieve zero-phase processing. The peak-to-peak values reach 20.33 µm and 18.15 µm for horizontal and vertical straightness, respectively, with corresponding standard deviations of 5.93 µm and 4.54 µm. This indicates that the QD output associated with the double-pass optical path, in which the beam is reflected by the CCR and returned to the measurement head, is significantly affected by environmental disturbances.

In contrast, for the single-pass optical path measured by QD2 and processed with the same filtering strategy, the peak-to-peak values of the horizontal and vertical straightness are reduced to 7.89 and 9.27 µm, respectively, whereas the corresponding standard deviations decrease markedly to 2.40 and 2.54 µm. Overall, QD2 demonstrated superior stability compared with QD throughout the entire measurement duration, exhibiting a lower noise amplitude and weaker long-term drift.

## 6. Single-Pass Common-Path Laser Beam Drift Digital Compensation Method

**Figure 8:**
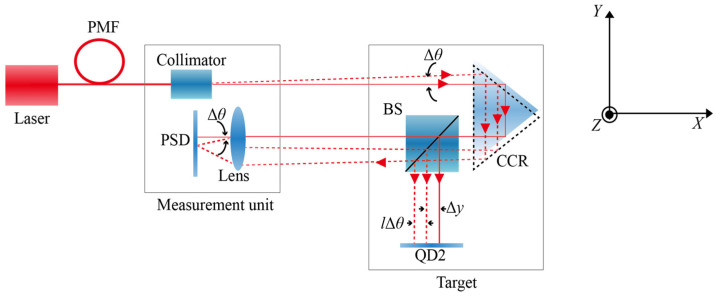
Schematic of the single-pass common-path digital compensation method for laser-beam drift.

Based on the experimental comparison results for the optical configuration shown in Figure 6, the straightness measurement in the single-pass optical path is significantly better than that in the double-pass optical path. Building on the common-path beam-drift compensation method previously proposed by our group, a digital beam-drift compensation approach for the single-pass common-path configuration is proposed, as shown in Figure 8. The measurement and reference beams propagate along the same optical path. When angular deflection occurs along the propagation path, the laser spot incident on a position-sensitive sensor (such as a QD or PSD) undergoes a corresponding positional shift due to the geometrical relationship of the optical path. Under single-pass conditions, the displacement increment on the sensor plane can be approximately expressed as

(7) $$\Delta y=l\tan\;\left(\Delta \theta \right)\approx l\Delta \theta ,$$

where Δ*θ* is the beam angular variation measured by the PSD (in radians). Compared with Equations (2) and (3), the contribution of the drift was reduced by a factor of two, highlighting the superiority of the single-pass optical path over the double-pass configuration.

### 6.1. Compensation of Mechanical Drift

**Figure 9:**
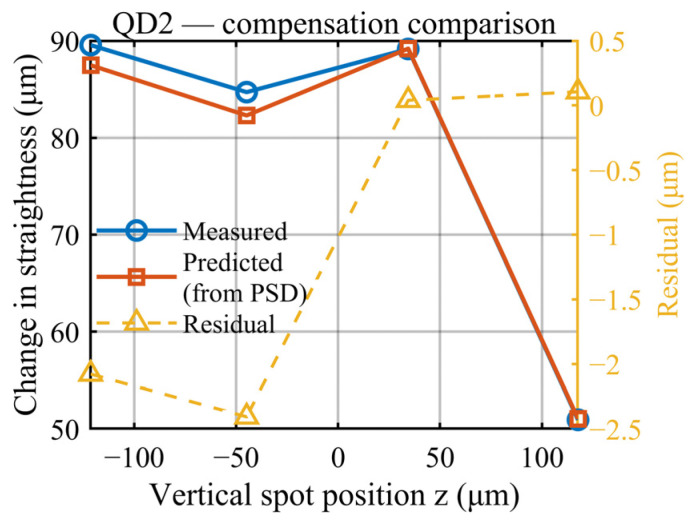
Results of mechanical drift compensation.

To verify the effectiveness of the single-pass common-path drift compensation method (Figure 8) in compensating for the mechanical drift, an experimental compensation platform was established. The measurement head and target mirror were fixed at opposite ends of the optical platform at a separation distance of 3.1 m. During the experiment, mechanical drift was simulated by intentionally varying the pitch angle of the measurement head. The angular variation was measured in real time using the PSD, and the displacement compensation value was calculated using Equation (7). This compensation value was then compared with the vertical straightness variation directly measured by QD2, and the residual between the two values was obtained, as shown in Figure 9.

Theoretically, this method can completely eliminate the straightness measurement errors induced by mechanical drift. The primary reasons for the residuals observed in Figure 8 are the relatively long optical path length in the experiment and the influence of external factors, such as atmospheric disturbances on the straightness measurement.

### 6.2. Compensation of Beam Drift Induced by Atmospheric Disturbances

**Figure 10:**
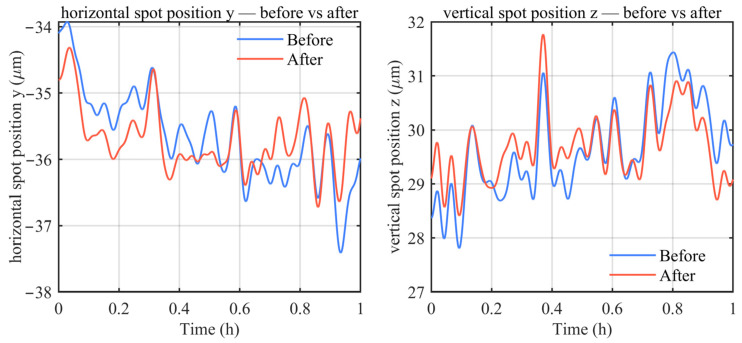
Comparison of experimental results for long-distance drift compensation.

To verify the effectiveness of the single-pass common-path drift compensation method shown in Figure 8 in compensating for measurement drift caused by air turbulence and other factors, an experimental setup was built under laboratory constraints, and experiments were carried out over a distance of 3.1 m. In the experiment, the measurement head and the target mirror were fixed at opposite ends of an optical platform with a separation distance of 3.1 m. Data were continuously acquired over a duration of 1 h, and the obtained results are presented in Figure 10.

As shown in Figure 10, in the horizontal direction, the peak-to-peak value is reduced from 3.48 µm before compensation to 2.41 µm after compensation, corresponding to a reduction of approximately 30.9%. The corresponding standard deviation decreases from 0.69 µm to 0.48 µm, also a reduction of approximately 30.9%. In the vertical direction, the peak-to-peak value decreases slightly from 3.63 µm to 3.36 µm, representing a reduction of approximately 7.3%, whereas the standard deviation is reduced from 0.81 µm to 0.62 µm, corresponding to a reduction of approximately 23.3%. These results demonstrate that the single-pass common-path drift compensation method can effectively suppress the low-frequency drift components and improve the stability of laser fiber-based collimation measurements.

## 7. Conclusions and Future Prospects

To address the degradation of measurement accuracy in long-distance laser straightness measurements caused by system calibration errors and air-disturbance-induced drift, this study proposes corresponding improvements from both calibration and compensation perspectives. First, a 2D calibration approach is adopted to effectively correct the nonlinear response and inter-axis coupling errors of the QD, thereby improving the calibration accuracy and reducing the system errors introduced during calibration. Second, through comparative simulations and experimental investigations of single- and double-pass optical paths, the measurement configuration was optimized and a single-pass common-path laser beam drift digital compensation method was proposed. This method effectively suppresses straightness measurement errors induced by mechanical drift and atmospheric turbulence. With these improvements, both the accuracy and stability of the long-distance laser straightness measurements were significantly enhanced, thereby providing an effective solution for high-precision straightness measurements over long distances.

## Data Availability

Data underlying the results presented in this paper are not publicly available at this time but may be obtained from the authors upon reasonable request.
